# Topical corticosteroid induced ulcerated striae^☆^^☆☆^

**DOI:** 10.1016/j.abd.2020.07.003

**Published:** 2020-11-18

**Authors:** Shyam B. Verma, Bhushan Madke

**Affiliations:** a Nirvan Skin Clinic, Makarpura, Vadodara, India; b Datta Meghe Institute of Medical Sciences (Deemed University), Jawaharlal Nehru Medical College, Sawangi Meghe, Wardha, India

**Keywords:** Adverse effects, Striae distensae, Topical steroid, Ulceration

## Abstract

We report four cases of ulcerated striae following misuse of fixed dose combinations creams containing clobetasol propionate with antifungal and antibacterial agents.

## Case report

**Figure 1:**
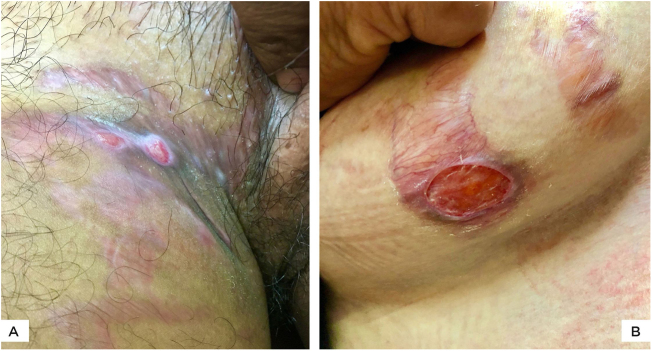
(A), Two superficial oval ulcers over striae in the inguinal region. (B), Beefy red oval ulcer over a wide atrophic stria with telangiectasia, extending from the anterior to the inferior aspect of breast. Active *tinea corporis* is observed.

**Figure 2:**
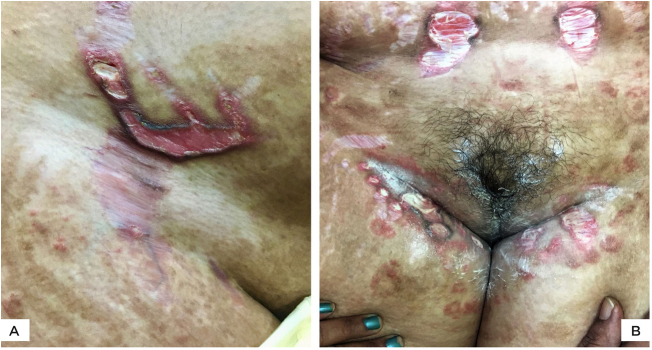
(A), Multiple, wide, beefy red, ulcerated striae over abdomen. Similar appearance, as well as linearly oriented oval ulcers in inguinal region. Widespread steroid-modified *tinea corporis* is observed. (B), Beefy red linear ulcers with hyperpigmented overhanging borders limited to the striae. Two superficial ulcerations on adjacent striae. *Tinea corporis* is observedin the background.

The authors report four cases of ulcerated striae following misuse of fixed-dose combinations (FDC) creams containing clobetasol propionate with antifungal and antibacterial agents. The present cases were overweight, between 27 − 40 years of age, and exhibited wide striae with secondary ulcerations. Three patients developed them after applying the implicated FDC creams for four months, five weeks, and five months, respectively (Figs. 1 A−B, 2A). The fourth patient (Fig. 2B) had preexisting striae following two pregnancies, many of which increased in size and ulcerated during the period of FDC application. Most ulcers were oval, some showing overhanging borders, whereas large ulcers frequently adopted the pattern of striae (Figs. 2 A−B). All ulcers were painful and were restricted to the striae. There was evidence of steroid-modified tinea corporis and history of erratic intake of oral antifungal drugs in all patients. Routine investigations were normal, including morning and evening serum cortisol levels. No biopsies were performed in view of the obvious clinical diagnosis and the possibility of delayed healing of TCS treated striae.

## Discussion

The literature on ulcerated striae (US) is scarce, and the majority of case reports implicate bevacuzimab, an anti-VEGF drug, used with high dose systemic corticosteroids, in the treatment of invasive brain tumors such glioblastomas.^1^, ^2^ Use of potent systemic or topical corticosteroids alone have also been implicated occasionally.^3^ Though not very common, the authors believe that the onset of US as a side effect of potent and super-potent topical steroids is underreported.^4^ Such creams are most frequently available as FDC, even over the counter, and are extensively misused in the treatment of superficial fungal infections in South Asia, Africa, and in many developing countries.^4^

The authors cite some old reports for their specific clinical relevance in the presents cases.^3^, ^5^, ^6^ Association of striae with steroid containing FDCs (triamcinolone, neomycin, gramicidin, or nystatin) dates back to 1963.^6^ Their predilection for warm, moist places prone to friction is known.^3^, ^5^, ^6^ They have been observed to occur as early as three weeks of application of TCS.^4^, ^5^, ^6^ Steroid-induced striae are wider than common SD. Some preexistent striae are known to enlarge upon application of TCS and new striae are observed to appear even after discontinuation of the cream.^4^, ^5^

Histopathological changes in striae include epidermal atrophy, loss of rete ridges, increased vascularity in striae rubra, less vascularity in striae alba, and dermal changes in extracellular matrix and fibrillary component of collagen and elastin.^6^, ^7^ However, the pathologic steps underlying ulceration in striae have not been elucidated. Interestingly, steroids are known to have anti-VEGF properties, similarly to bevacizumab.^8^ While twelve cases of bevacizumab in combination with systemic steroids are reported in the English literature, both drugs individually can cause ulceration.^3^, ^9^, ^10^ The authors hypothesize that long-term application of a potent topical steroid such as clobetasol may lead to slow ischemia, on account of its vasoconstrictive and anti-VEGF properties, leading to ulceration.^8^, ^9^ The well-documented features of profound epidermal atrophy, high degree of vascularity, limited elasticity, and reduced tensile strength of steroid induced striae reinforce this hypothesis.

In conclusion, the authors presented four cases of patients with *tinea corporis* and US induced by FDCs containing clobetasol propionate, which are widely misused in India. The authors believe this is an underreported side effect of the FDCs that are fueling the epidemic-like situation of tinea corporis in India. Drug policy makers need to regulate permissions to manufacture and sell such hazardous and often irrational creams. It is also time for agencies such as the World Health Organization to look into this menace. Finally, the mechanism of ulceration of steroid induced striae needs to be further studied and elucidated.

## Financial support

None declared.

## Authors’ contributions

Shyam B. Verma: Approval of the final version of the manuscript; preparation and writing of the manuscript; manuscript critical review.

Bhushan Madke: Preparation and writing of the manuscript; critical literature review; manuscript critical review.

## Conflicts of interest

None declared.
